# Complicated Acute Appendicitis Conservatively Treated in Two Patients With Crohn's Disease: The Role of Intestinal Ultrasound and Contrast‐Enhanced Ultrasound

**DOI:** 10.1002/jcu.70288

**Published:** 2026-06-17

**Authors:** Cataletti Giovanni, Gridavilla Daniele, Nylund Kim, Colombo Francesco, Danelli Piergiorgio, Maconi Giovanni

**Affiliations:** ^1^ Gastroenterology Unit, Department of Biomedical and Clinical Sciences “L. Sacco” Hospital, University of Milano Milan Italy; ^2^ National Centre for Ultrasound in Gastroenterology, Haukeland University Hospital Bergen Norway; ^3^ Department of Clinical Medicine University of Bergen Bergen Norway; ^4^ Division of General Surgery, Department of Biomedical and Clinical Sciences “L. Sacco” Hospital, University of Milano Milan Italy

**Keywords:** acute appendicitis, contrast‐enhanced ultrasound, Crohn's disease, intestinal ultrasound

## Abstract

According to the most recent guidelines, uncomplicated acute appendicitis could be treated conservatively using broad‐spectrum antibiotics, but if radiological signs of appendix rupture are present, appendectomy should be considered as first‐line treatment. There is limited evidence in literature regarding the non‐operative management of complicated acute appendicitis, particularly in the subset of patients with Crohn's disease. Intestinal and contrast‐enhanced ultrasound appear pivotal to both diagnosing complications and promptly identifying the non‐responding patients. We describe two cases of complicated acute appendicitis, successfully treated with broad‐spectrum antibiotic therapy and tight monitoring with both intestinal and contrast‐enhanced ultrasound, in patients with Crohn's disease.

## Graphical abstract

Intestinal ultrasound and contrast‐enhanced ultrasound were used to diagnose, monitor, and guide conservative treatment of complicated acute appendicitis in two patients with Crohn's disease. Serial imaging enabled early identification of treatment response, allowing long‐term surgery‐free outcomes.

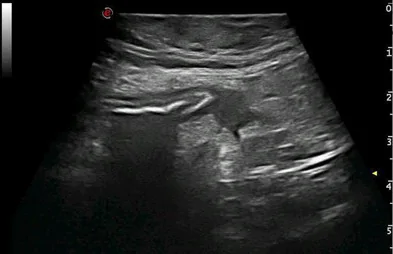

## 1 Introduction

Acute appendicitis represents a common cause of acute abdominal pain and the most common disease leading to emergency abdominal surgery worldwide (Dimitrios Moris et al. 2021). The incidence of acute appendicitis in western countries, range from 5.7 to 50 per 100.000 inhabitants per year, with a peak between 10 and 30‐years (Di Saverio et al. 2020).

The diagnosis of acute appendicitis is based on medical history, physical examination, symptoms and signs, laboratory evaluation and radiological findings, mainly intestinal ultrasound (IUS), and computed tomography (CT). The latter are usually pivotal to classify it as uncomplicated, when there is no evidence of rupture, and complicated if associated with abscess or phlegmon (Dimitrios Moris et al. 2021).

In the last years, contrast‐enhanced ultrasound (CEUS) has proven high sensitivity and specificity in detecting and differentiating between intra‐abdominal phlegmon and abscesses in several gastrointestinal conditions, such as inflammatory bowel diseases and acute appendicitis; therefore this technique could be used to both diagnose and follow‐up patients with these conditions instead of CT‐scan (Ripollés et al. 2013; Gokli et al. 2021).

The optimal management strategy for acute appendicitis remains a topic of ongoing debate, particularly in patients with Crohn's disease, where surgical intervention carries increased risks. The latest guidelines of the World Society of Emergency Surgery (WSES), drafted by Di Saverio et al. (Di Saverio et al. 2020), affirm that non‐operative management with broad‐spectrum antibiotics and percutaneous drainage is a reasonable first‐line treatment for patients with acute appendicitis complicated by phlegmon or abscess, especially in settings where laparoscopic expertise is not available. On the other hand, very recent guidelines of the Society of American Gastrointestinal and Endoscopic Surgeons (SAGES), drafted by Kumar et al. (Kumar et al. 2024), state that adult and pediatric patients with complicated acute appendicitis should be managed operatively. Furthermore, they suggest that also patients with uncomplicated acute appendicitis should undergo either delayed (> 12 h) or immediate (< 12 h) operation (Kumar et al. 2024). However, there are specific situations in which a conservative (non‐operative) approach may be considered, especially if surgery has a high risk of actual or future complications. In fact, in patients with significant cecal inflammation, longer symptom duration/delayed presentation, or a well‐formed abscess, greater consideration should be given to initial non‐operative management to minimize surgical risks (Kumar et al. 2024).

## 2 Methods

This study retrospectively analyzed two patients with complicated acute appendicitis and Crohn's disease, focusing on clinical progression, imaging findings, and response to antibiotic therapy. In these cases, surgery was not preferred as the first option, due to the high risk of complications and the possibility of unnecessary resection (Patel et al. 2016). No signs of sepsis or peritonitis were present at baseline in both patients. After the diagnostic intestinal ultrasound (IUS) and contrast‐enhanced ultrasound (CEUS), performed at admission, broad‐spectrum antibiotics were started. The antibiotic regimen selected (piperacillin/tazobactam and ciprofloxacin plus metronidazole) was active against gram‐negative bacteria and anaerobic organisms, the most involved in acute appendicitis complicated by abscess (Dimitrios Moris et al. 2021). Concomitant immunosuppressive drugs (adalimumab in one patient) were temporarily discontinued. During the hospitalization, the patients underwent tight follow‐up with daily physical examination, biochemical tests every 48–72 h, and IUS 5–7 days after antibiotics start, in order to promptly identify signs of non‐response to therapy. Follow‐up visits with IUS and CEUS were performed at 1 and 3 weeks after discharge.

## 3 Case Presentations and Results

### 3.1 Case 1

A 43‐year‐old man, with colonic Crohn's disease since 2019, was referred to the Emergency Department for moderate abdominal pain in the right iliac region and fever. Medical history was negative but for Crohn's disease, in stable clinical remission for 4 years, under treatment with adalimumab 40 mg every 2 weeks.

At baseline, laboratory findings showed elevated C‐reactive protein (CRP; 78 mg/L; normal value < 10 mg/L) and white blood cell count (14 700/μl). On physical examination, there was tenderness in the right iliac fossa, directly and indirectly when palpating in the lower left quadrant, but no rebound tenderness was observed. The CT scan of the abdomen and an intestinal ultrasound (IUS) coupled with intravenous (IV) contrast enhancement (CEUS) confirmed the presence of acute appendicitis, complicated by an abscess with a maximum diameter of 2 cm (Figures 1 and 2 and Video S1). Nevertheless, IUS demonstrated normal bowel wall thickness and echo‐pattern in both the colon and terminal ileum, without color‐Doppler signal (modified Limberg score 0) or medium‐contrast enhancement in these segments. Therefore, no evidence of Crohn's disease reactivation was observed.

**FIGURE 1:**
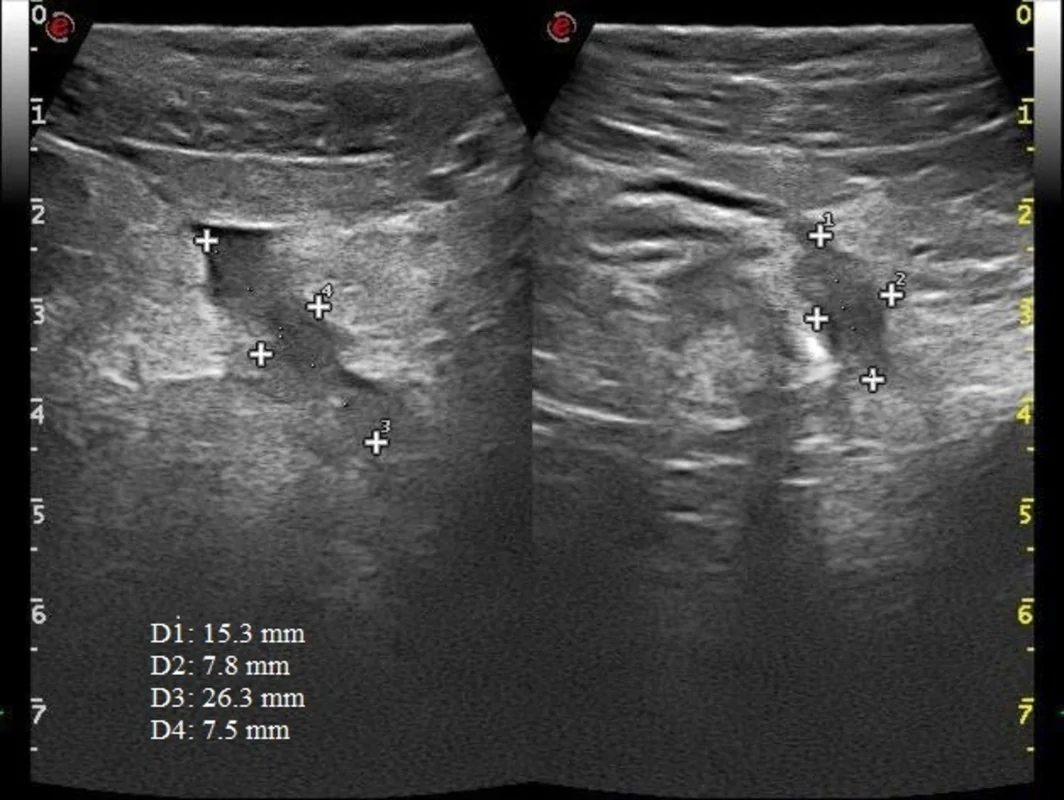
Ultrasound image showing a hypoechoic area in longitudinal (left side) and transverse (right side) view, close to vermicular appendix.

**FIGURE 2:**
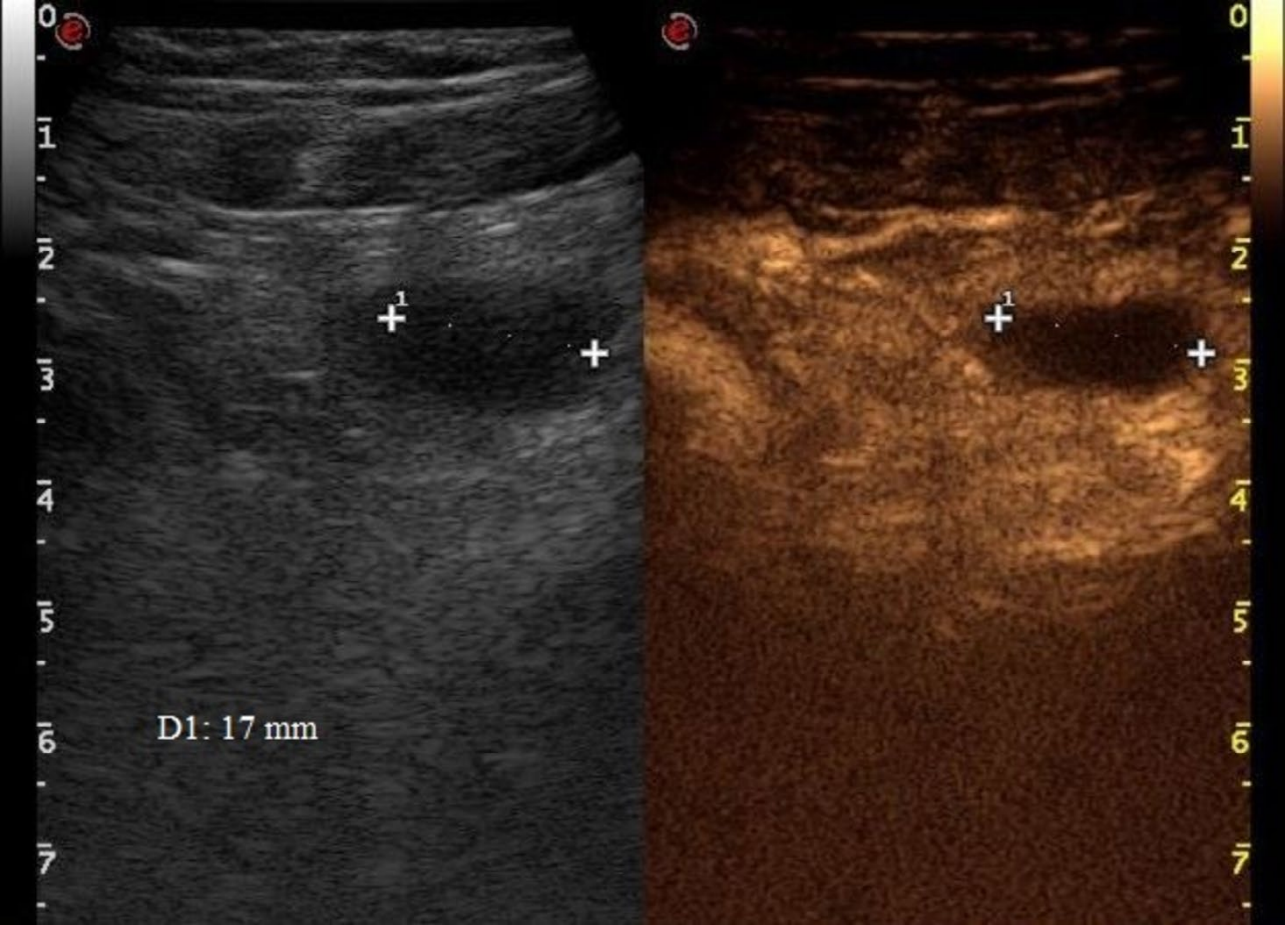
Contrast‐enhanced ultrasound showing a peri‐appendicular hypoechoic area of about 2 cm of diameter, not enhanced after intravenous medium contrast injection (right side of the image). Findings suggestive for peri‐appendicular abscess.

Because of both concomitant biologic therapy and long‐standing clinical remission, following a multidisciplinary discussion with the surgeons and radiologists, it has been decided to postpone surgery and start broad‐spectrum antibiotic therapy with piperacillin/tazobactam 4.5 g every 6 h intravenously. adalimumab was temporarily discontinued.

After 10 days, we observed complete regression of abdominal pain and marked improvement of inflammatory markers (CRP 3 mg/L). piperacillin/tazobactam was discontinued, while orally administered ciprofloxacin was started (500 mg twice daily) and continued after the discharge.

At 1‐week and 3‐week follow‐up visits, we perform IUS and CEUS that showed the reduction in size of the peri‐appendicular abscess up to 8 mm. Furthermore, at 3‐week follow‐up, white blood cells and CRP showed normal values. Considering the favorable outcome of the lesion and the normalization of biochemical tests, ciprofloxacin administration was discontinued.

Two months after the onset of symptoms, abdominal ultrasound examination showed a normal appendix without evidence of abscess or phlegmon in the peri‐appendicular area and adalimumab was reintroduced. The patient remained asymptomatic and did not require surgical intervention during the five‐year follow‐up period.

### 3.2 Case 2

A 46‐year‐old man came to the emergency department for fever, chills and epigastric pain radiating to the right lumbar and right iliac region. The patient had uncomplicated ileal Crohn's disease diagnosed in 2018, in long‐standing clinical remission without immunomodulatory or biological therapy, and cholelithiasis. He was taking mesalamine 3 g per day for symptomatic benefit.

The physical examination showed marked tenderness on palpation in the right lumbar and right iliac fossa. No rebound tenderness was present at the physical examination.

Laboratory findings showed a marked increase in CRP (169 mg/L; normal value < 10 mg/L) and white blood cells count (11 500/μl).

CT scan and IUS showed a vermicular appendix with a diameter of 19 mm and thickened (> 3 mm) and hyperemic walls coupled with hypertrophy of meso‐appendix, with a small fluid collection that was further confirmed by CEUS (Figures 3, 4, 5 and Video S2). IUS showed normal findings in the colon and only a prominent hyperechoic submucosal layer in the terminal ileum, without bowel wall thickening (up to 2.0 mm; normal value < 3.0 mm) or color‐Doppler signal (modified Limberg score 0). No evidence of Crohn's disease reactivation or penetrative complications (e.g., fistula) was observed.

**FIGURE 3:**
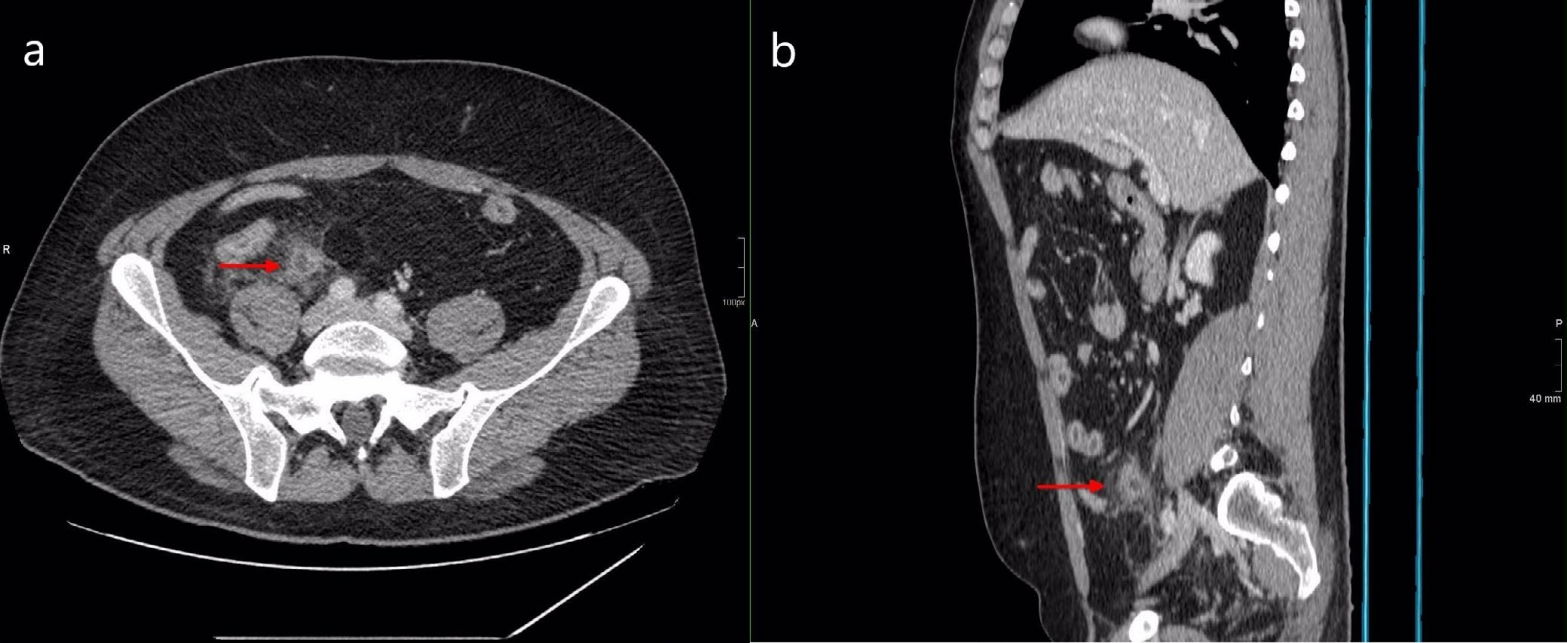
CT‐scan showing in axial (a) and sagittal (b) view, the presence of inhomogeneity of the peri‐appendicular fat associated with thickening of appendicular wall (red arrows). Findings suggestive for acute appendicitis.

**FIGURE 4:**
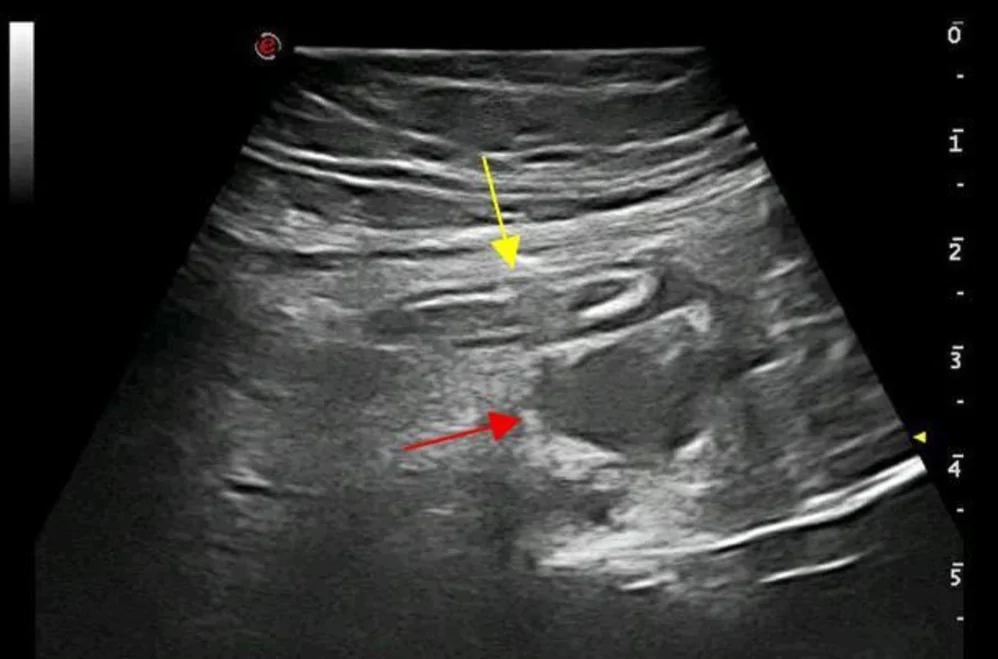
Ultrasound image showing the vermicular appendix (yellow arrow) with thickened wall and, just behind (deeper), the presence of a hypoechoic area suggestive for inflammatory phlegmon or abscess (red arrow).

**FIGURE 5:**
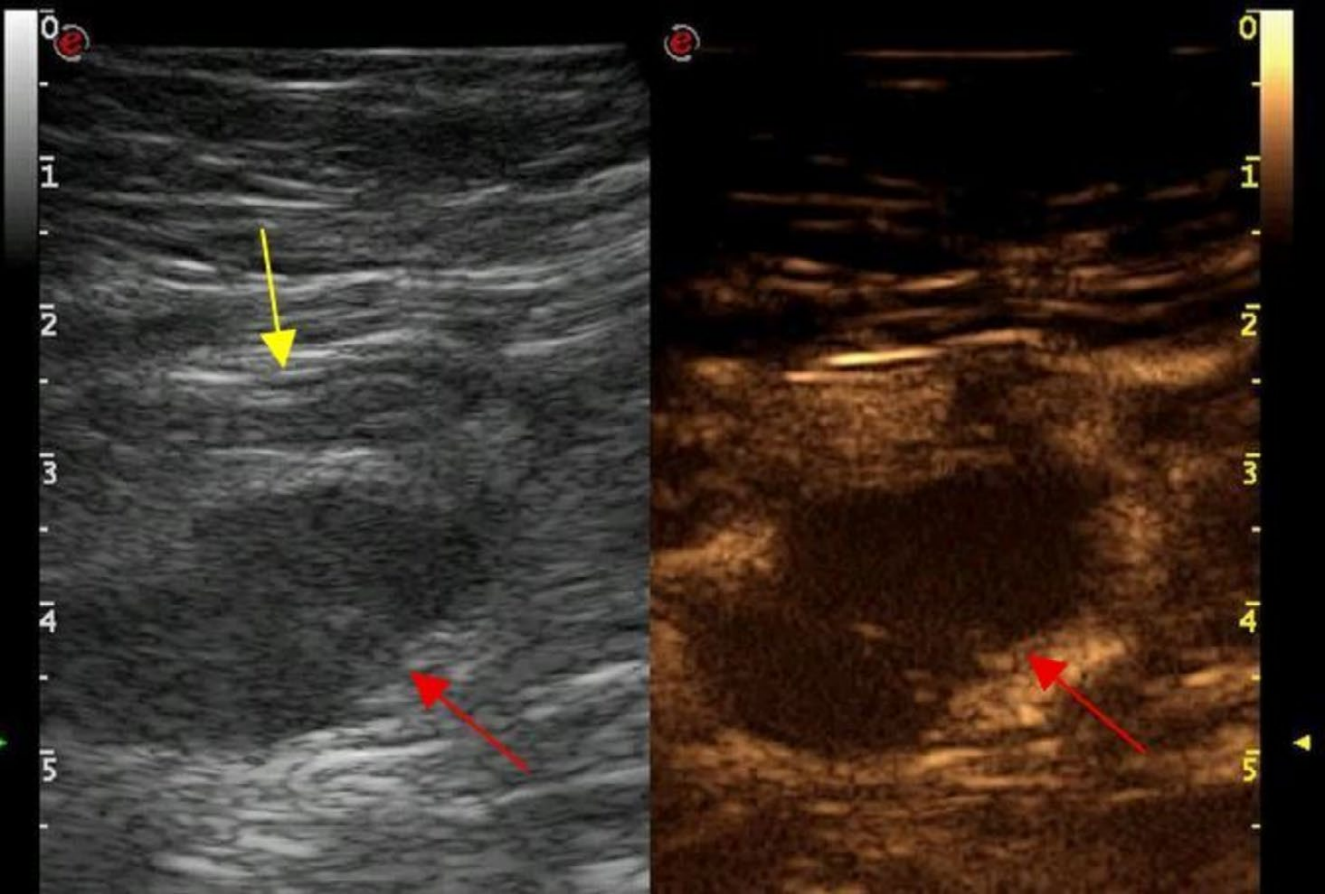
Contrast‐enhanced ultrasound showing a hypoechoic area (red arrow) very close to the vermicular appendix (yellow arrow on the left side of the image), not enhanced after intravenous medium contrast injection (right side of the image). Findings suggestive for peri‐appendicular abscess.

The patient was treated with a broad‐spectrum antibiotic therapy with ciprofloxacin 500 mg twice daily and metronidazole 500 mg three times daily. After 4 days, we observed a complete regression of abdominal pain and improvement of inflammatory markers (CRP 116 mg/L). The patient was discharged with tight clinical and sonographic monitoring.

An IUS performed 1 week later showed the normalization of the diameter of the appendix and its wall thickness, but persistence of a hypoechoic area of 20 mm diameter, which at CEUS showed an almost complete enhancement (Figures 6 and 7 and Video S3) suggesting the healing of abscess collection. The following IUS and CEUS performed after 3 weeks showed a progressive reduction in size of the peri‐appendicular inflammatory mass and the normalization of the CRP. The antibiotic therapy was therefore withheld. The patient remained asymptomatic and did not require surgical intervention during the four‐year follow‐up period.

**FIGURE 6:**
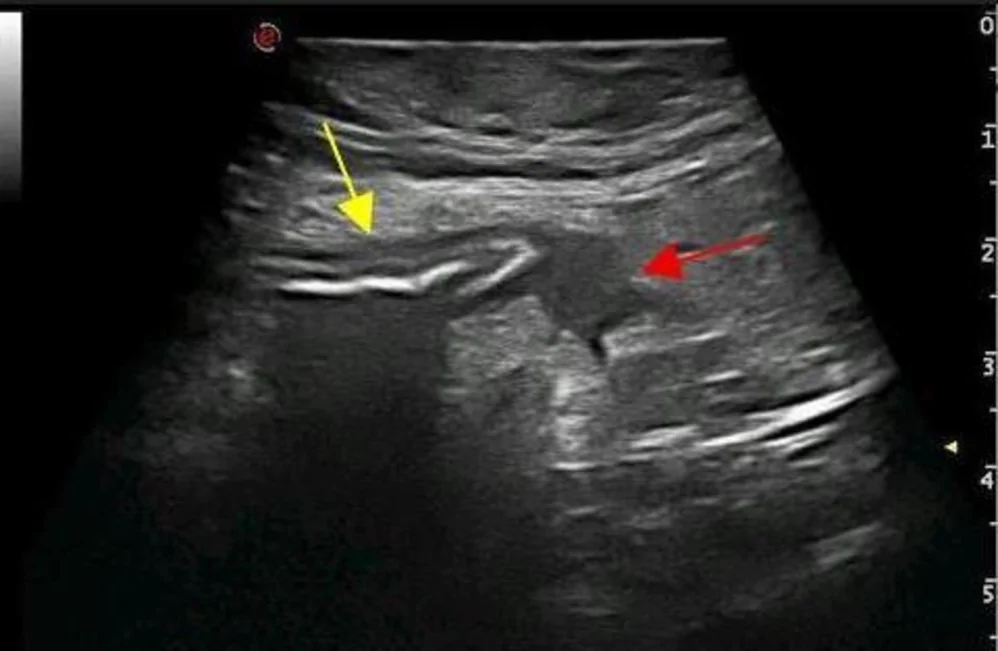
1‐week follow‐up ultrasound, showing reduction in size of the hypoechoic area (red arrow) near the vermicular appendix (yellow arrow) after antibiotic therapy.

**FIGURE 7:**
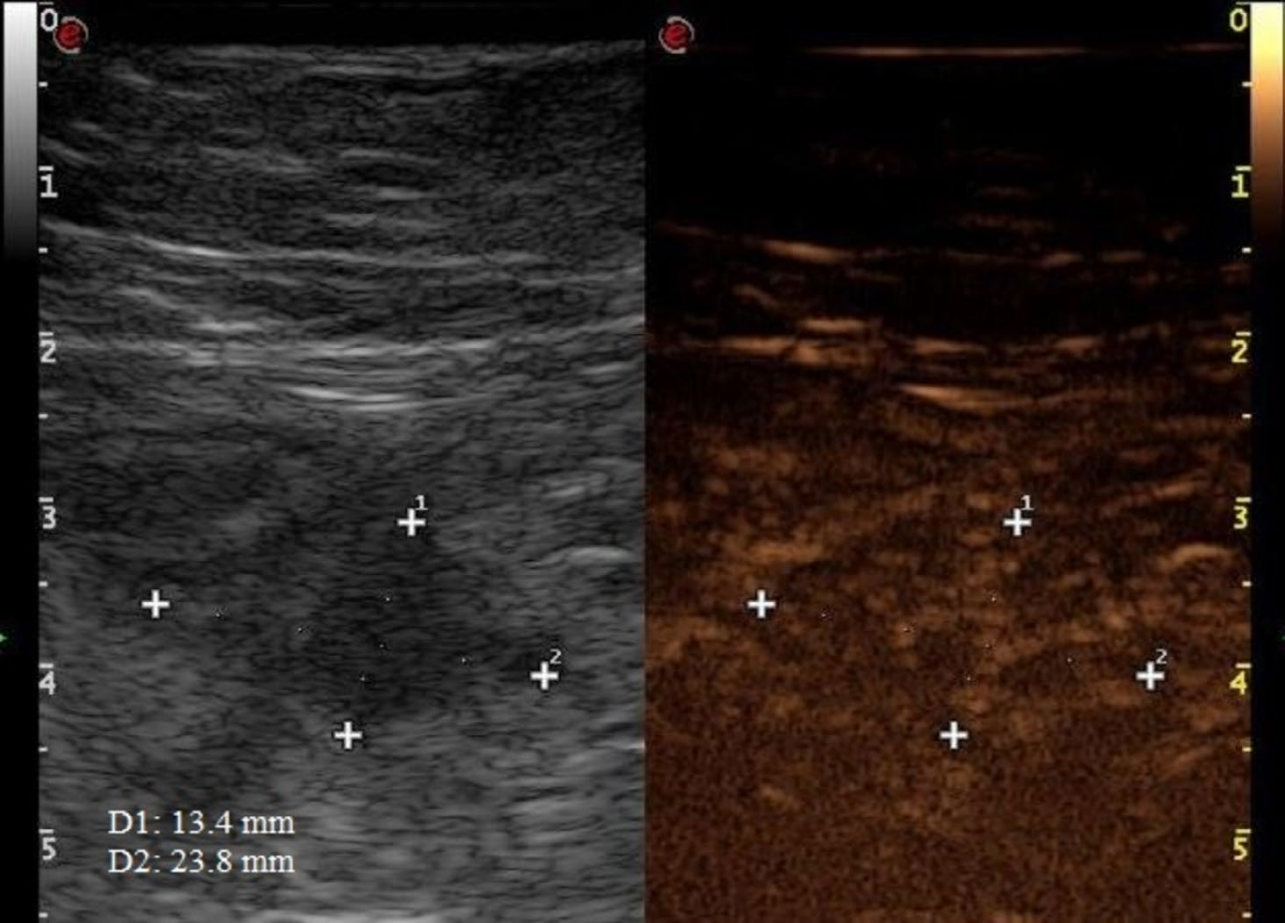
1‐week follow‐up with CEUS, showing complete enhancement of the hypoechoic area after intravenous medium contrast injection (right side of the image). Findings suggestive for resolution of peri‐appendicular abscess.

## 4 Discussion

Growing evidence in the last years is demonstrating that antibiotic treatment could be a feasible and safe alternative to appendectomy for patients with uncomplicated acute appendicitis (Dimitrios Moris et al. 2021; Di Saverio et al. 2020; Doleman et al. 2024; Scheijmans et al. 2025).

On the other hand, the most appropriate management of complicated acute appendicitis is still debated. As mentioned above, while the latest WSES guidelines affirm that non‐operative management with broad‐spectrum antibiotics and percutaneous drainage is a reasonable first‐line treatment for patients with complicated acute appendicitis (Di Saverio et al. 2020), very recent SAGES guidelines state that both adult and pediatric patients affected by complicated acute appendicitis should be managed operatively, except in patients with significant cecal inflammation, longer symptom duration, delayed presentation, or a well‐formed abscess, in order to minimize surgical risks (Kumar et al. 2024).

In this setting, for patients with Crohn's disease, some concerns should be highlighted. Particularly, patients with acute appendicitis and also affected by ileal or colonic Crohn's disease could be under treatment with immunosuppressants (corticosteroids or biologic therapy) chronically or during flares, thus representing a risk factor for post‐surgical infections or other complications (Patel et al. 2016; Billioud et al. 2013; Rosenfeld et al. 2013; Yang et al. 2014). This is particularly true for corticosteroids that should be tapered whenever possible before surgery, to reduce the risk of postoperative septic complications, intra‐abdominal abscesses and anastomotic leak (Adamina et al. 2024). On the contrary, the latest ECCO guidelines suggest to continue biologic drugs administration before surgery, as current evidence shows that preoperative treatment with anti‐TNF agents, vedolizumab and ustekinumab does not increase the risk of postoperative complications in patients with Crohn's disease undergoing abdominal surgery (Adamina et al. 2024).

Anyway, regardless of the therapy used, the effective non‐operative management of acute complicated appendicitis allows for prompt reintroduction of immunosuppressive drug after clinical, radiological, and biochemical disease resolution.

It is pivotal to highlight that up to 70% of Crohn's disease patients require abdominal surgery during their life for disease's flares or development of complications (stenosis, *fistulas* and abscesses) (Patel et al. 2016). In addition, some of them are at higher risk of postsurgical complications, also after appendectomy, especially in case of ileal involvement (Machado et al. 2010).

According to that, *Machado et al*. (Machado et al. 2010) described a case of enterocutaneous *fistula* developed after appendectomy alone (instead of ileocecal resection) in a patient with unsuspected ileocecal Crohn's disease misdiagnosed as complicated acute appendicitis. For these reasons, it would be preferable to avoid unnecessary abdominal surgery in such patients whenever possible. In fact, in such cases, ileocecal resection instead of appendectomy alone should be the preferred strategy, thus leading to unnecessary ileal resection in most cases.

Furthermore, avoiding unnecessary abdominal surgery in patients affected by Crohn's disease is also pivotal to preventing the formation of surgical adhesions that can make future surgery more difficult or challenging.

The possibility of avoiding surgery, by using successful conservative management with antibiotic therapy, has also been suggested in patients with complicated acute diverticulitis (Garfinkle et al. 2016), thus showing that in the absence of acute peritonitis, conservative management of simple septic complication is feasible.

In fact, Garfinkle et al. (Garfinkle et al. 2016) published a retrospective study in which they describe 73 patients with acute diverticulitis complicated by abscess managed non‐operatively. Of these, only 9 patients underwent subsequent surgery, 7 (9.6%) because of complicated recurrence requiring elective sigmoidectomy, and 2 (2.7%) because of a recurrent episode with peritonitis. According to that, the authors concluded that after initial successful non‐operative management of diverticulitis with abscess, expectant management with non‐operative intent is a safe long‐term option with low rates of surgery, especially in the emergency setting.

Intestinal ultrasound (IUS) can play a major role in differentiating complicated acute appendicitis from reactivation of Crohn's disease complicated by fistula and/or abscess. Indeed, this imaging technique allows the exclusion of inflammatory involvement of the cecum, colon, or terminal ileum, as indicated by findings such as bowel wall thickening, loss of normal stratification of the bowel wall echo pattern, and increased color Doppler signals in the affected intestinal segment. It also enables the exclusion of features of penetrating disease, such as fistulas originating from the bowel wall. For these reasons, IUS appears pivotal in guiding subsequent patient management.

Furthermore, IUS can detect signs of acute appendiceal inflammation, including an outer appendiceal diameter greater than 6 mm, non‐compressibility of the inflamed appendix, increased vascularity of the appendiceal wall on color Doppler imaging, and loss of wall stratification in cases of gangrenous appendicitis. In such cases, careful assessment of the surrounding area is essential to identify additional features of acute appendicitis, such as hyperechoic peri‐appendiceal fat, mesenteric lymphadenopathy, and the presence of complex fluid collections around the appendix (i.e., abscesses) (Dirks et al. 2019).

It is important to note that both inflammatory mass (phlegmon) and abscess may appear as hypoechoic areas surrounding the inflamed appendix, and differentiation between these two conditions may not be reliably achieved using greyscale ultrasound alone.

In such clinical scenarios, CEUS could be a very useful tool to differentiate inflammatory mass (phlegmon) from abscesses, and to monitor the treatment response (Ripollés et al. 2013; Gokli et al. 2021; Maconi et al. 2018). After the injection of intravenous medium contrast agent (sulfur hexafluoride microbubbles) at the dosage of 2.5–5.0 mL (20 μg–40 μg), preferably with a high frequency linear transducer (5–15 MHz), it is possible to study the peri‐appendicular area and well differentiate inflammatory masses (phlegmons) characterized by wide enhancement of the medium contrast from abscesses, in which there is no enhancement of the medium contrast.

In conclusion, with this report we confirm that in patients with Crohn's disease and complicated acute appendicitis presenting with stable clinical condition and no evidence of sepsis or peritonitis, non‐operative management with broad‐spectrum antibiotics may be a viable option with positive long‐term results.

In these cases, the antibiotics used should be active against gram‐negative bacteria and anaerobic organisms. The best regimen is not clearly defined, but the most used are ertapenem or piperacillin/tazobactam in monotherapy, and third‐generation cephalosporin plus metronidazole or fluoroquinolones plus metronidazole as combination therapy (Dimitrios Moris et al. 2021).

Anyway, we feel that close monitoring of the lesion, using appropriate tools like IUS and CEUS, is mandatory to clearly define the size and volume of fluid collection, providing an accurate baseline assessment of the lesion before antibiotic therapy, to monitor the response and promptly decide whether the patient should undergo surgical appendectomy in case of inadequate response.

In view of this, in the absence of adequate expertise in the use of intestinal ultrasound and contrast‐enhanced ultrasound, it could be safer to suggest initial surgical management (especially if laparoscopic expertise is available) for patients presenting with complicated acute appendicitis and Crohn's disease, because of the impossibility to promptly identify the non‐responders and send them to surgery before developing more severe complications, such as peritonitis or sepsis.

## 5 Conclusions

Laparoscopic appendectomy is the first‐line therapy for acute appendicitis complicated by appendiceal rupture, with subsequent abscess or phlegmon formation. Nevertheless, certain cases of complicated acute appendicitis may be managed conservatively, provided there is careful patient selection and close clinical and radiological monitoring. Therefore, if patients are in stable clinical condition, with localized symptoms and without signs of peritonitis or sepsis, a non‐operative approach could be considered. Furthermore, in patients with Crohn's disease, conservative management presents several advantages such as avoiding unnecessary intestinal resection, reducing the risk of post‐surgical complications and preventing the formation of surgical adhesions that can make future surgery more challenging. For these reasons, in this subset of patients, a non‐operative approach could be a viable option, with positive long‐term results. In this setting, a close follow‐up with IUS and CEUS should be performed in order to promptly identify the non‐responding patients that require abdominal surgery. Further prospective studies, with larger cohorts of patients, should substantiate our findings regarding non‐operative management of complicated acute appendicitis in Crohn's disease and the potential role of IUS and CEUS in this clinical scenario.

## Author Contributions

Conceptualization: C.G. and G.D.; Methodology: C.G., G.D., and M.G.; Validation: M.G., C.F., and D.P.; Investigation: C.G., G.D., and M.G.; Resources: C.G. and N.K.; Data curation: C.G. and M.G.; Writing – original draft preparation: C.G. and G.D.; Writing – review and editing: C.G., M.G. and N.K.; Visualization: C.F. and D.P.; Supervision: M.G. All authors have read and agreed to the published version of the manuscript.

## Funding

The authors have nothing to report.

## Ethics Statement

The study was conducted according to the guidelines of the Declaration of Helsinki, and ethical approval was not required by our Ethics Committee for reporting human case series (< or = 5).

## Consent

Informed consent was obtained from all subjects involved in the study.

## Conflicts of Interest

The authors declare no conflicts of interest.

## Supporting information


**Video S1:** CEUS showing the absence of contrast enhancement in the peri‐appendicular hypoechoic area, supporting the diagnosis of peri‐appendicular abscess.

**Video S2:** CEUS showing the absence of contrast enhancement in a peri‐appendicular hypoechoic area originating from the vermicular appendix.

**Video S3:** 1‐week follow‐up CEUS showing complete enhancement of the hypoechoic area after intravenous medium contrast injection.

## Data Availability

The data that support the findings of this study are available on request from the corresponding author. The data are not publicly available due to privacy or ethical restrictions.
